# Willingness of individuals with Li-Fraumeni syndrome to participate in a cancer prevention trial: a survey study

**DOI:** 10.1007/s10689-023-00339-y

**Published:** 2023-06-23

**Authors:** Farina J. Struewe, Sarah Schott, Martina de Zwaan, Christian P. Kratz

**Affiliations:** 1 Pediatric Hematology and Oncology, Hannover Medical School, Hannover, Germany; 2 Department of Gynecology and Obstetrics, University Hospital Heidelberg, Heidelberg, Germany; 3 Department of Psychosomatic Medicine and Psychotherapy, Hannover Medical School, Hannover, Germany

Li-Fraumeni syndrome (LFS;OMIM151623) is a cancer prone condition that is caused by pathogenic germline variants in the *TP53* tumor suppressor gene. LFS is associated with a high and life-long elevated risk of developing a range of brain and non-brain solid tumors as well as hematologic neoplasms. In addition, LFS is a common known genetic cause of cancer. For example, > 1% of cases of childhood cancer are associated with a pathogenic germline variant in *TP53* and similar numbers are being observed in adults [1, 2]. Due to the high cancer risk and the clinically relevant frequency of occurrence of the condition, the development of cancer preventative strategies for *TP53* pathogenic variant carriers represents an urgent scientific task. A rising number of *TP53* pathogenic variant carriers are being identified through the increasing use of next generation sequencing methods in clinical medicine.

A promising compound that is being tested as a cancer preventative agent in individuals with LFS is metformin, a type 2 diabetes drug that activates AMP-activated protein kinase (AMPK) and suppresses mammalian target of rapamycin (mTOR) [3]. A meta-analysis demonstrated that patients with type 2 diabetes taking metformin have a significantly lower cancer risk [4]. Moreover, metformin delayed the emergence of cancer in a LFS murine model [5]. To assess the feasibility of a metformin prevention trial in individuals with LFS, we have addressed the question whether LFS individuals and parents of affected children are willing to participate or would enroll an affected child in a randomized cancer prevention trial with metformin.

We conducted a German-wide, exploratory questionnaire-based study with 121 self-designed items and two established questionnaires on quality of life (QoL) (Supplement 1). The questionnaire included a scenario of a randomized cancer prevention trial with metformin. We also included the SF-12, an instrument for recording the generic health-related QoL. It consists of 12 questions resulting in two scale scores: the Physical Component Summary (PCS-12) and the Mental Component Summary (MCS-12). Both are standardized combined scores with a mean of 50 and a standard deviation of 10 based on data from the US general population with the higher score indicating better QoL. Fear of progression (FoP) was assessed with the 12‐item Fear of Progression Questionnaire Short Form (PA‐F‐SF) for patients. Higher scores indicate higher FoP. Values ≥ 34 are being regarded as critical.

We recruited via social media for 51 days. Recruitment was conducted via the LFS registry and the German LFS association using various online channels and newsletters. We created an online video, in which the study was explained. The questionnaire was available online via the SoSciSurvey portal (program version 3.2.40). We asked adults ≥ 18 years with LFS and parents of children with LFS for their demographic data, experience with and attitudes toward study participation**.** Results were analyzed via descriptive statistics. Ethics approval was obtained prior to initiation of the trial.

The Table 1 depicts recruitment and composition of the cohort. The Fig. 1 summarizes major results and additional results are provided in Supplement 2. The unbalanced female: male ratio of 7:1 may be due to the fact that women with breast cancer, the most common LFS-associated tumor in females, are frequently undergoing genetic testing. The majority of individuals with LFS were willing to participate in a randomized metformin cancer prevention trial. Willingness to enroll the own child with LFS in such a trial was high in the majority of parents of a child with LFS. There were no differences found between responses given by individuals with *versus* without children. Altruistic factors as well as own health advantages were identified as main motivating factors for participation. The most common discouraging aspects concerning study participation were fear of side effects by a study medication, followed by study design aspects like use of placebo or randomization. QoL in terms of physical and mental health was below average. T-scores were 47.9 (22.2–65.1, SD ± 9.9) and 43.4 (15.0–60.1, SD ± 10.9), respectively. We observed a high fear of progression (mean score 37.2, SD ± 9.7, cut-off 34).

**Table 1:** Recruitment and composition of the cohort

Study population	
Informed consent provided	161
Completion of last item	120/161
Mean age	41.4 years (19 to 75 years, SD 10.9)
Gender	Females: 105/120 (87.5%) Males: 15/120 (12.5%)
Diagnosed with LFS	112/120 (93.3%)
History of at least 1 malignancy	88/120 (88.3%)
Participants with children	81/120 (76.5%)
Children with LFS	42/81 (51.9%)
Children with at least 1 malignancy	18/81 (22.2%)
Children that died of cancer	4/18 (22.2%)
Participants that lost at least 1 parent during childhood	29/120 (24.2%)

**Fig. 1:**
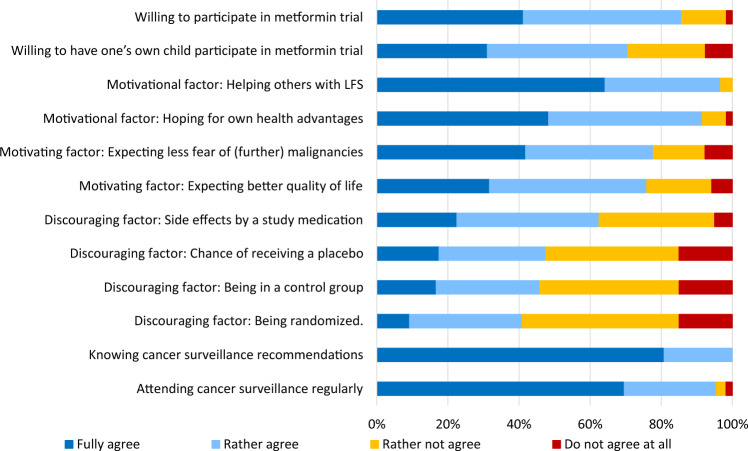
Major results of questionnaire based feasibility trial

Willingness to participate in a cancer prevention trial with metformin is high among patients with LFS, which makes recruitment for an efficacy study with metformin feasible. We found factors that influence this willingness and gained insight into LFS patients’ attitudes toward cancer prevention and clinical trials in general. These data can be used to inform future studies. An appropriate characterization of nonresponders to ensure that nonresponse bias does not threaten the validity of the findings is impossible due to the recruitment via social media and this represents a limitation of this survey study.

### Supplementary Information

Below is the link to the electronic supplementary material.

Supplementary file1 (DOCX 48 KB)

Supplementary file2 (DOCX 31 KB)

## Data Availability

All de-identified data are available in the supplement.
