# Supplementary material for: Willingness of individuals with Li-Fraumeni syndrome to participate in a cancer prevention trial: a survey study
Source: Fam Cancer. 2023 Jun 23;22(4):495–7. doi: 10.1007/s10689-023-00339-y (PMC10541338; doi:10.1007/s10689-023-00339-y)
Supplement: Supplementary file 1 — Supplementary file1 (DOCX 48 KB) [file 10689_2023_339_MOESM1_ESM.docx]

Supplement 1. Questionnaire employed in the study

1. Informed consent to study participation:

- I hereby consent to participate in the questionnaire study and agree to the analysis of the data I have entered.

2. I agree to be contacted again by the study team in future if necessary.

- Yes
- No

3. Please, give us an e-mail address where we may contact you if necessary.

Please make sure that the spelling is correct. In case of typing errors we will not be able to contact you.

- Free text: *enter your mail address here*

4. In the following, we will generate a code to which your data will be linked. However, the code does not allow third parties to trace back your personal data more precisely.

Please enter the required numbers/letters below.

- First letter of your mother's first name: *1 letter*
- First letter of your father's first name: *1 letter*
- Month of birth of your mother: *number with two digits (e.g. 03 for March)*
- Birth month of your father: *number with two digits (e.g. 03 for March)*
- Your gender: *1 letter (w=female / m=male / d=divers)*
- Your year of birth: *number with two digits* *(e.g. 81 for 1981)*

Thank you for taking the time to complete our questionnaire!

Please read the questions carefully and answer all of them. The questions are supposed to generate an assessment of the willingness to participate in clinical trials among affected individuals with Li-Fraumeni Syndrome and parents of affected children. In the further course, we have always abbreviated Li-Fraumeni Syndrome as "LFS".

5. Have you been diagnosed with Li-Fraumeni Syndrome?

- Yes
- No

6. How many children do you have?

- no children
- 1 child
- 2 children
- 3 children
- 4 children
- more children, free text: *Please enter the number of your children here*

Cancer risk / Surveillance / Cancer Prevention

Below, we would like to assess your attitudes towards cancer prevention in Li-Fraumeni Syndrome (LFS). Prevention/delay of the occurrence of cancer is differed from detection of cancer that has already occurred as early as possible. For individuals with LFS, recommendations on examinations for early cancer detection are established. However, LFS-specific measures to prevent the occurrence of cancer do not yet exist, so far. There is one drug that is being studied in research to see if it can reduce the risk of cancer in LFS. This drug, metformin, has been used to date in the treatment of diabetes. Since the cancer-reducing effect has not yet been proven, metformin should not be taken outside of trials because otherwise its effect could possibly not be shown in future.

Please rate whether the following statements about cancer prevention in LFS apply for you.

7. I am aware of the surveillance program specifically recommended for individuals with LFS.

- Fully applies
- Rather applies
- Does rather not apply
- Does not apply at all

8. I regularly attend the surveillance exams specifically recommended for individuals with LFS.

- Fully applies
- Rather applies
- Does rather not apply
- Does not apply at all

9. I consider the surveillance program specifically recommended for individuals with LFS to be unnecessary.

- Fully applies
- Rather applies
- Does rather not apply
- Does not apply at all

10. I am afraid of the results of the surveillance exams specifically recommended for individuals with LFS.

- Fully applies
- Rather applies
- Does rather not apply
- Does not apply at all

11. The waiting times for the surveillance exams specifically recommended for individuals with LFS are too long.

- Fully applies
- Rather applies
- Does rather not apply
- Does not apply at all

12. Travel distances to surveillance exams specifically recommended for individuals with LFS are too far.

- Fully applies
- Rather applies
- Does rather not apply
- Does not apply at all

13. On a scale of 0 to 100%, how high do you rate your personal cancer risk?

- 0 %
- 10 %
- 20 %
- 30 %
- 40 %
- 50 %
- 60 %
- 70 %
- 80 %
- 90 %
- 100 %

14. I estimate my personal cancer risk to be higher than in the average population.

- Fully applies
- Rather applies
- Does rather not apply
- Does not apply at all

If you do not have children, please still answer the questions concerning children. In that case, please choose the answer that you would most like to give if you had children.

15. Preventing cancer is an important issue for me.

- Fully applies
- Rather applies
- Does rather not apply
- Does not apply at all

16. I estimate that cancer prevention is more important for me than for the average population.

- Fully applies
- Rather applies
- Does rather not apply
- Does not apply at all

17. I would make use of recommended measures to prevent cancer in LFS.

- Fully applies
- Rather applies
- Does rather not apply
- Does not apply at all

18. Cancer prevention in LFS should start as early as possible, even in childhood.

- Fully applies
- Rather applies
- Does rather not apply
- Does not apply at all

19. Cancer prevention in LFS should not begin until adulthood.

- Fully applies
- Rather applies
- Does rather not apply
- Does not apply at all

20. If the drug metformin was approved for adults with LFS to prevent cancer, I would take it.

- Fully applies
- Rather applies
- Does rather not apply
- Does not apply at all

21. If the drug metformin was approved for children with LFS to prevent cancer, I would let my affected child take it.

- Fully applies
- Rather applies
- Does rather not apply
- Does not apply at all

22. If there was another approved medication for adults with LFS to prevent cancer, I would take it.

- Fully applies
- Rather applies
- Does rather not apply
- Does not apply at all

23. If there was another approved medication for children with LFS to prevent cancer, I would let my child take it.

- Fully applies
- Rather applies
- Does rather not apply
- Does not apply at all

Previous experience with clinical trials

In the following, we ask you to answer some questions about your previous experience with clinical trials. A clinical trial is a study that requires written consent from the participants. As a rule, it serves to collect medical data for research purposes, e.g. to gain knowledge about examination or treatment methods for certain patient groups. A distinction is made between observational studies, in which data are collected only, and intervention studies, in which participants receive a drug or take part in examinations, for example. Recommendations for medical care can be made on the basis of study results.

In the following, the term "trial" refers to medical studies. How would you rate your knowledge of the following about research and trials? Please choose if the statements apply to you.

I feel well informed concerning…

24. ... research and trials in general.

- Fully applies
- Rather applies
- Does rather not apply
- Does not apply at all

25. ... trials with adult participants.

- Fully applies
- Rather applies
- Does rather not apply
- Does not apply at all

26. ... trials with children participating.

- Fully applies
- Rather applies
- Does rather not apply
- Does not apply at all

27. ... the use of a placebo in medical trials.

- Fully applies
- Rather applies
- Does rather not apply
- Does not apply at all

28. ... the concept of randomization.

- Fully applies
- Rather applies
- Does rather not apply
- Does not apply at all

29. ... considering cost-effectiveness of participation in a trial.

- Fully applies
- Rather applies
- Does rather not apply
- Does not apply at all

30. ... the opportunity to quit participation before the end of a trial without having disadvantages.

- Fully applies
- Rather applies
- Does rather not apply
- Does not apply at all

Which sources have you used so far to get information on medical trials? Please choose the statements that apply to you.

So far, I have...

31. ... used information provided by media / internet / journals.

- Yes
- No

32. ... received information from family members / friends.

- Yes
- No

33. ... received information from my local doctor(s) (e. g. general practitioner).

- Yes
- No

34. ... received information from my doctor(s) in hospital.

- Yes
- No

35. ... evaluated these references as trustful.

- Fully applies
- Rather applies
- Does rather not apply
- Does not apply at all

Which of the following sources would you use in order to get information about clinical trials concerning LFS/cancer? Please choose if the statements apply for you.

I would search for information about clinical trials via…

36. ... clinic websites.

- Fully applies
- Rather applies
- Does rather not apply
- Does not apply at all

37. ... the FIT-Website (www.krebs-praedisposition.de).

- Fully applies
- Rather applies
- Does rather not apply
- Does not apply at all

38. ... the LFSA-Website (Li-Fraumeni Syndrome Association) or contact the LFSA Deutschland e. V.

- Fully applies
- Rather applies
- Does rather not apply
- Does not apply at all

39. ... support groups other than the LSFA.

- Fully applies
- Rather applies
- Does rather not apply
- Does not apply at all

40. ... social media (Facebook, Instagram, ...).

- Fully applies
- Rather applies
- Does rather not apply
- Does not apply at all

41. ... printed prochures or journals.

- Fully applies
- Rather applies
- Does rather not apply
- Does not apply at all

42. ... local doctors.

- Fully applies
- Rather applies
- Does rather not apply
- Does not apply at all

43. ... hospital doctors.

- Fully applies
- Rather applies
- Does rather not apply
- Does not apply at all

In the following we would like you to let us know about your family members‘ /relatives’ attitude towards medical trials.

44. Do you have family members / relatives?

- Yes
- No

45. Do you have family members / relatives who know that you /your child have/has been diagnosed with Li-Fraumeni Syndrom?

- Yes
- No

How is your family members’ / relatives’ attitude towards LFS?

My family members…

46. ... are interested and research information concerning LFS.

- Fully applies
- Rather applies
- Does rather not apply
- Does not apply at all

47. ... take care and support LFS affected family members.

- Fully applies
- Rather applies
- Does rather not apply
- Does not apply at all

48. ... would probably also participate in this survey or other medical trials.

- Fully applies
- Rather applies
- Does rather not apply
- Does not apply at all

Which experience have you already gained by participating in trials (before this survey)? Please evaluate, if the following statements apply for you. The term “trial” is meant in the context of medical trials (surveys, examinations, registry, therapy …).

49. I have been offered the participation in a trial or had the opportunity to participate, respectively.

- Yes
- No

50. More than once I have been offered the participation in a trial or had the opportunity to participate, respectively.

- Yes
- No

51. I have already participated in a trial.

- Yes
- No

52. I have already participated in a trial more than once.

- Yes
- No

53. My child/children has/have been offered the participation in a trial or had the opportunity to participate, respectively.

- Yes
- No

54. More than once my child/children has/have been offered the participation in a trial or had the opportunity to participate, respectively.

- Yes
- No

55. My child/children has/have already participated in a trial.

- Yes
- No

56. My child/children has/have already participated in a trial more than once.

- Yes
- No

57. I know the cancer predisposition syndrome registry (CPS-registry Hannover/Heidelberg).

- Yes
- No

58. I know the whole-body-MRI study für affected with LFS in Heidelberg/Hannover.

- Yes
- No

59. I know the survey on psychosocial aspects in LFS in Heidelberg or Hannover.

- Yes
- No

60. I am registered in the cancer predisposition syndrome registry (CPS-registry Hannover/Heidelberg).

- Yes
- No

61. My child/children is/are registered in the cancer predisposition syndrome registry (CPS-registry Hannover/Heidelberg).

- Yes
- No

62. I have already participated in the whole-body-MRI study für affected with LFS in Heidelberg/Hannover.

- Yes
- No

63. I have already participated in the survey on psychosocial aspects in LFS in Heidelberg or Hannover.

- Yes
- No

64. In general, I would evaluate my experience with trials as „good“.

- Fully applies
- Rather applies
- Does rather not apply
- Does not apply at all

65. I would approve cancer risk trials with adults with LFS.

- Fully applies
- Rather applies
- Does rather not apply
- Does not apply at all

66. I would approve cancer risk trials with children with LFS.

- Fully applies
- Rather applies
- Does rather not apply
- Does not apply at all

67. I believe that cancer risk studies with adults with LFS are too burdensome for them.

- Fully applies
- Rather applies
- Does rather not apply
- Does not apply at all

68. I believe that cancer risk studies with children with LFS are too burdensome for them.

- Fully applies
- Rather applies
- Does rather not apply
- Does not apply at all

Factors influencing the willingness to participate in clinical/medical trials

The following statements describe your potential willingness to participate in medical trials. Please, evaluate whether they apply for you. There are also statements involving your children. In case you do not have any children, please still answer the questions. You can choose the answer you imagine to choose if you had children, then. In case you are a parent of a child with LFS but you have not been diagnosed LFS yourself, we would also like you to answer all questions. For LFS-specific questions, you can choose the answer you imagine to choose if you had LFS, then. In the following, the term „trial“ stands for medical trials.

69. In principle, I would participate in medical trials in general.

- Fully applies
- Rather applies
- Does rather not apply
- Does not apply at all

70. In principle, I would participate in a LFS trial.

- Fully applies
- Rather applies
- Does rather not apply
- Does not apply at all

71. In principle, I would let my child participate in medical trials in general.

- Fully applies
- Rather applies
- Does rather not apply
- Does not apply at all

72. In principle, I would let my child participate in a LFS trial.

- Fully applies
- Rather applies
- Does rather not apply
- Does not apply at all

I would rather participate in a trial, if…

73. ... I would receive an expense allowance for it.

- Fully applies
- Rather applies
- Does rather not apply
- Does not apply at all

74. ... the trial’s aim would be to investigate a new medication’s effect in LFS.

- Fully applies
- Rather applies
- Does rather not apply
- Does not apply at all

75. ... the trial’s aim would be to investigate the tolerance of a new medication in LFS.

- Fully applies
- Rather applies
- Does rather not apply
- Does not apply at all

76. ... in the trial a new medication would be prescribed with the aim to decrease cancer risk in LFS.

- Fully applies
- Rather applies
- Does rather not apply
- Does not apply at all

77. … it was conducted internationally.

- Fully applies
- Rather applies
- Does rather not apply
- Does not apply at all

78. ... it would include coming to the clinics for study visits.

- Fully applies
- Rather applies
- Does rather not apply
- Does not apply at all

79. ... it would include additional blood collection.

- Fully applies
- Rather applies
- Does rather not apply
- Does not apply at all

80. ... I would be randomized to one out of two treatment options.

- Fully applies
- Rather applies
- Does rather not apply
- Does not apply at all

81. ... it would be possible that I receive a placebo.

- Fully applies
- Rather applies
- Does rather not apply
- Does not apply at all

82. ... it would be possible that I would be part of a control group that does not receive the investigated medication.

- Fully applies
- Rather applies
- Does rather not apply
- Does not apply at all

83. ... I could have side effects from a medication I would take.

- Fully applies
- Rather applies
- Does rather not apply
- Does not apply at all

In the following, factors that might influence your willingness to participate in a trial are shown. Please evaluate whether the statements apply for you.

84. I believe that I might receive a better treatment in a LFS trial.

- Fully applies
- Rather applies
- Does rather not apply
- Does not apply at all

85. I think that my participation in a trial might help others who are affected with LFS.

- Fully applies
- Rather applies
- Does rather not apply
- Does not apply at all

86. I think I could contribute to medical improvements in LFS.

- Fully applies
- Rather applies
- Does rather not apply
- Does not apply at all

87. I would feel like a guinea-pig.

- Fully applies
- Rather applies
- Does rather not apply
- Does not apply at all

88. I trust doctors of a trial’s team.

- Fully applies
- Rather applies
- Does rather not apply
- Does not apply at all

89. I am afraid of possible side-effects from medication that is being investigated in a trial.

- Fully applies
- Rather applies
- Does rather not apply
- Does not apply at all

90. I would regret having participated in case the medication would not have the expected effect.

- Fully applies
- Rather applies
- Does rather not apply
- Does not apply at all

91. I think that research only supports the investigators‘ carrier.

- Fully applies
- Rather applies
- Does rather not apply
- Does not apply at all

92. I imagine to be supervised more closely during participation in a trial.

- Fully applies
- Rather applies
- Does rather not apply
- Does not apply at all

93. My family would support my participation in a trial.

- Fully applies
- Rather applies
- Does rather not apply
- Does not apply at all

94. I am afraid of health-related disadvantages if I do not participate in an offered trial.

- Fully applies
- Rather applies
- Does rather not apply
- Does not apply at all

95. I would quit participation prematurely in case I found out that I would be randomized to the control group that does not receive the study medication.

- Fully applies
- Rather applies
- Does rather not apply
- Does not apply at all

Scenario: medication for cancer prevention in LFS

In the following we describe a scenario of a cancer prevention trial for affected individuals with LFS. Please, read the scenario carefully.

Scenario: A trial is supposed to investigate whether the medication metformin can reduce cancer risk in individuals with LFS. Children and adults diagnosed with LFS are allowed to participate in the trial. Participation is only possible, if metformin has not been taken before. Although metformin is already approved for diabetes treatment, it still remains unclear whether it reduces cancer risk in LFS. During the trial, metformin is supposed to be taken by the participants daily. There might be side-effects which are expected to be dissolved by dose reductions or stop of intake. In order to evaluate metformin’s effect, the participants are divided into two groups, from which one receives the medication and the other one does not. The group allocation is random and without the option to choose. The trial runs for 5 years. Additionally, participation includes occasional blood collections and yearly visits in the trial center. On basis of the trial’s findings, it will be decided whether the medication will be approved for cancer risk reduction for all people affected with LFS.

Because the cancer risk reducing effect of metformin in LFS has not been proven, yet, it is important not to take the medication outside of trials for now. This could lead to the fact that its effect cannot be shown.

The following statements refer to your potential willingness to participate in the trial described in the scenario above. Please evaluate if the statements apply for you. There are also statements involving your children. In case you do not have any children, please still answer the questions. You can choose the answer you imagine to choose if you had children, then. In case you are a parent of a child with LFS but you have not been diagnosed LFS yourself, we would also like you to answer all questions. For LFS-specific questions, you can choose the answer you imagine to choose if you had LFS, then

96. In principle, I would participate in the above-mentioned trial in the scenario.

- Fully applies
- Rather applies
- Does rather not apply
- Does not apply at all

97. I would wish children to participate in the trial in the scenario as well, so that – in the case of positive results - the medication could also be approved for children.

- Fully applies
- Rather applies
- Does rather not apply
- Does not apply at all

98. In principle, I would let my child participate in the above-mentioned trial in the scenario.

- Fully applies
- Rather applies
- Does rather not apply
- Does not apply at all

In the following, factors that might influence your willingness to participate in the trial in the scenario are shown. Please evaluate whether the statements apply for you.

In principle, I would participate in the trial in the above-described scenario…

99. ... after having received more information about the study medication.

- Fully applies
- Rather applies
- Does rather not apply
- Does not apply at all

100. ... because I would hope for health advantages for myself.

- Fully applies
- Rather applies
- Does rather not apply
- Does not apply at all

101. ... because I might help other people with LFS by participating.

- Fully applies
- Rather applies
- Does rather not apply
- Does not apply at all

102. ... because I think that my quality of life might improve.

- Fully applies
- Rather applies
- Does rather not apply
- Does not apply at all

103. ... because I think that by participating I might have less fear of (further) occurence malignancies.

- Fully applies
- Rather applies
- Does rather not apply
- Does not apply at all

104. ... although the study medication might cause side-effects.

- Fully applies
- Rather applies
- Does rather not apply
- Does not apply at all

105. ... after being explained advantages and risks of participation.

- Fully applies
- Rather applies
- Does rather not apply
- Does not apply at all

I would not participate in the trial in the above-mentioned scenario…

106. ... because I am afraid of potential side-effects.

- Fully applies
- Rather applies
- Does rather not apply
- Does not apply at all

107. ... because I would not want to be in the control group at all.

- Fully applies
- Rather applies
- Does rather not apply
- Does not apply at all

108. ... if I would not receive an expense allowance.

- Fully applies
- Rather applies
- Does rather not apply
- Does not apply at all

109. ... because I would not want to undergo blood collections.

- Fully applies
- Rather applies
- Does rather not apply
- Does not apply at all

110. ... because the way to the study center would be too time-consuming.

- Fully applies
- Rather applies
- Does rather not apply
- Does not apply at all

111. ... because I think that my quality of life would suffer from it.

- Fully applies
- Rather applies
- Does rather not apply
- Does not apply at all

112. ... because I think that my health would suffer from it.

- Fully applies
- Rather applies
- Does rather not apply
- Does not apply at all

Health/Condition

In the following we have further questions concerning your health status an your condition.

In the following questions, your health status will be assessed. Der Bogen ermöglicht es, im Zeitverlauf nachzuvollziehen, wie Sie sich fühlen und wie Sie im Alltag zurechtkommen. Bitte beantworten Sie jede der Fragen, indem Sie ankreuzen, was am besten auf Sie zutrifft.

113. In general, would you say your health is

- excellent
- very good
- good
- fair
- poor

The following questions are about activities you might do during a typical day. Does your health now limit you in these activities? If so, how much?

114. moderate activities such as moving a table, pushing a vacuum cleaner, bowling or playing golf:

- yes, limited a lot
- yes, limited a little
- no, not limited at all

115. climbing several flights of stairs

- yes, limited a lot
- yes, limited a little
- no, not limited at all

During the past 4 weeks, have you had any of the following problems with your work or other regular daily activities as a result of your physical health?

116. Accomplished less than you would like.

- Yes
- No

117. Were limited in the kind of work or other activities.

- Yes
- No

During the past 4 weeks, have you had any of the following problems with your work or other regular daily activities as a result of any emotional problems (such as feeling depressed or anxious)?

118. Accomplished les than you would like.

- Yes
- No

119. Did work or activities less carefully as usual.

- Yes
- No

120. During the past 4 weeks, how much did pain interfere with your normal work (including work outside

- Not at all
- A little bit
- Moderately
- Quite a lot
- Extremely

These questions are about how you have been feeling during the past 4 weeks. For each question, please give the one answer that comes closest to the way you have been feeling.

How much of the time during the past 4 weeks...

121. ... have you felt calm and peaceful?

- All of the time
- Most of the time
- A good bit oft he time
- Some of the time
- A little of the time
- None of the time

122. ... did you have a lot of energy?

- All of the time
- Most of the time
- A good bit oft he time
- Some of the time
- A little of the time
- None of the time

123. ... have you felt down-hearted and blue?

- All of the time
- Most of the time
- A good bit oft he time
- Some of the time
- A little of the time
- None of the time

124. During the past 4 weeks, how much of the time has your physical health or emotional problems interfered with your social activities (like visiting friends, relatives, etc.)?

- All of the time
- Most of the time
- A good bit oft he time
- Some of the time
- A little of the time
- None of the time

Below you will see a list of statements that are related to your illness and possible future concerns. Some questions will not apply to you. Please make a mark under “never” in these cases.

125. I become anxious if I think my disease may progress.

- Never
- Seldom
- Sometimes
- Often
- Very often

126. I am nervous prior to doctors’ appointments or periodic examinations.

- Never
- Seldom
- Sometimes
- Often
- Very often

127. I am afraid of pain.

- Never
- Seldom
- Sometimes
- Often
- Very often

128. I have concerns about reaching my professional goals because of my illness.

- Never
- Seldom
- Sometimes
- Often
- Very often

129. When I am anxious, I have physical symptoms such as a rapid heartbeat, stomach ache or agitation.

- Never
- Seldom
- Sometimes
- Often
- Very often

130. The possibility of my children contracting my disease disturbs me.

- Never
- Seldom
- Sometimes
- Often
- Very often

131. It disturbs me that I may have to rely on strangers for activities of daily living.

- Never
- Seldom
- Sometimes
- Often
- Very often

132. I am worried that at some point in time I will no longer be able to pursue my hobbies because of my illness.

- Never
- Seldom
- Sometimes
- Often
- Very often

133. I am afraid of severe medical treatments during the course of my illness.

- Never
- Seldom
- Sometimes
- Often
- Very often

134. I worry that my treatment could damage my body.

- Never
- Seldom
- Sometimes
- Often
- Very often

135. I worry about what will become of my family if something should happen to me.

- Nie
- Selten
- Manchmal
- Oft
- Sehr oft

136. The thought that I might not be able to work due to my illness disturbs me.

- Never
- Seldom
- Sometimes
- Often
- Very often

At last, we would like you to give us some general information on your demographics. Therefore, please answer the following questions:

137. When have you been diagnosed with Li-Fraumeni Syndrome?

Please select the according calender year. [Bitte auswählen] (1990 bis 2022)

138. When are you born?

- [please select] (1942 to 2004)

139. You are…

- female
- male
- diverse

140. Which is you highest educational degree?

- No school degree
- German „Sonderschulabschluss“Special school leaving certificate
- German “Haupt-/Volkshochschulabschluss”
- German “Realschulabschluss” / Middle school degree
- German „Fachhochschulreife“
- German „Fachabitur“
- Vocational training
- High school diploma
- University degree
- Other, free text: Please enter your highest educational qualification yourself

141. What is your employment status right now?

- school/university/training
- unemployed/seeking employment
- self-employed
- housewife/houseman
- employee
- employed in public service / civil servant
- retired/pensionered
- Other, free text: Please enter your employment status yourself

142. Has one of your children been diagnosed with cancer?

- No
- Yes, one child has/had at least one malignancy
- Yes, more than one child has/had at least one malignancy each

143. Have you ever been diagnosed with cancer?

- Yes
- No

144. Have your children been diagnosed with Li-Fraumeni Syndrome?

- No
- Yes, 1 child
- Yes, 2 children
- Yes, 3 children
- Yes, 4 children
- Yes, more than 4 children, free text: please enter the amount of your children diagnosed with LFS

145. Do you smoke?

- No
- Yes
- In the past yes, not anymore

146. How often do you drink alcohol?

- Never
- Less than once per month
- Once per month
- 2 to 4 times per montht
- 2 to 3 times per week
- 4 times or more per week

147. Have you lost a parent to cancer when you were younger than 18 years?

- Yes
- No

148. How many siblings do you have?

- No siblings
- 1
- 2
- 3
- More than 3, free text: Please enter the amount of your siblings

149. How many of your siblings have been diagnosed with Li-Fraumeni Syndrome?

- None
- 1
- 2
- 3
- More than 3, free text: Please enter the amount of your siblings with LFS

150. Haso one of your children died from cancer?

- Yes
- No
