# Supplementary material for: Willingness of individuals with Li-Fraumeni syndrome to participate in a cancer prevention trial: a survey study
Source: Fam Cancer. 2023 Jun 23;22(4):495–7. doi: 10.1007/s10689-023-00339-y (PMC10541338; doi:10.1007/s10689-023-00339-y)
Supplement: Supplementary file 2 — Supplementary file2 (DOCX 31 KB) [file 10689_2023_339_MOESM2_ESM.docx]

Supplement 2. Additional Results of Survey Study

| Category/Question | Reply No. (%) | | | |
| --- | --- | --- | --- | --- |
| Self-estimated cancer risk |  |  |  |  |
|  | Fully applies | Rather applies | Does rather not apply | Does not apply at all |
| I estimate my personal cancer risk to be higher than in the average population. | 99 (71.2%) | 34 (25.5%) | 4 (2.9%) | 2 (1.4%) |
| Lifestyle - Risk factors / Cancer surveillance |  |  |  |  |
|  | Fully applies | Rather applies | Does rather not apply | Does not apply at all |
| I am aware of the surveillance program specifically recommended for individuals with LFS. | 113 (81.3%) | 26 (18.7%) | 0 (0.0%) | 0 (0.0%) |
| I regularly attend the surveillance exams specifically recommended for individuals with LFS. | 90 (69.8%) | 34 (26.4%) | 3 (2.3%) | 2 (1.6%) |
|  | yes |  | no |  |
| Do you smoke? | 13 (12.4%) |  | 92 (87.6%) |  |
| Attitudes toward cancer prevention |  |  |  |  |
|  | Fully applies | Rather applies | Does rather not apply | Does not apply at all |
| Preventing cancer is an important issue for me. | 116 (85.3%) | 19 (14.0%) | 1 (0.7%) | 0 (0.0%) |
| Cancer prevention in LFS should start as early as possible, even in childhood. | 107 (78.7%) | 23 (16.9%) | 6 (4.4%) | 0 (0.0%) |
| Cancer prevention in LFS should not begin until adulthood. | 1 (0.7%) | 8 (5.9%) | 28 (20.6%) | 99 (72.8%) |
| I would make use of recommended measures to prevent cancer in LFS. | 107 (84.3%) | 18 (14.2%) | 2 (1.6%) | 0 (0.0%) |
| If the drug metformin was approved for adults with LFS to prevent cancer, I would take it. | 78 (61.4%) | 40 (31.5%) | 8 (6.3%) | 1 (0.8%) |
| If there was another approved medication for adults with LFS to prevent cancer, I would take it. | 73 (57.9%) | 47 (37.3%) | 6 (4.8%) | 0 (0.0%) |
| If the drug metformin was approved for children with LFS to prevent cancer, I would let my affected child take it. | 60 (44.1%) | 53 (39.0%) | 21 (15.4%) | 2 (1.5%) |
| If there was another approved medication for children with LFS to prevent cancer, I would let my child take it. | 58 (42.6%) | 58 (42.6%) | 19 14.0% | 1 (0.7%) |
| I consider the surveillance program specifically recommended for individuals with LFS to be unnecessary. | 0 (0.0%) | 1 (0.7%) | 13 (9.4) | 125 (89.9%) |
| I am afraid of the results of the surveillance exams specifically recommended for individuals with LFS. | 39 (28.1%) | 56 (40.3%) | 30 (21.6%) | 14 (10.1%) |
| The waiting times for the surveillance exams specifically recommended for individuals with LFS are too long. | 10 (7.2%) | 31 (22.3%) | 74 (53.2%) | 24 (17.3%) |
| Travel distances to surveillance exams specifically recommended for individuals with LFS are too far. | 16 (11.5%) | 47 (33.8%) | 50 (36.0%) | 26 (18.7%) |
| Knowledge about clinical trials |  |  |  |  |
| I feel well informed concerning… | Fully applies | Rather applies | Does rather not apply | Does not apply at all |
| ... research and trials in general. | 33 (24.8%) | 55 (41.1%) | 40 (30.1%) | 5 (3.8%) |
| ... trials with adult participants. | 33 (24.8%) | 49 (36.8%) | 46 (34.6%) | 5 (3.8%) |
| ... trials with children participating. | 15 (11.3%) | 22 (16.5%) | 59 (44.4%) | 37 (27.8%) |
| ... the use of a placebo in medical trials. | 43 (32.3%) | 37 (27.8%) | 32 (24.1%) | 21 (15.8%) |
| ... the concept of randomization. | 33 (25.0%) | 36 (27.3%) | 27 (20.5%) | 36 (27.3%) |
| ... considering cost-effectiveness of participation in a trial. | 34 (25.6%) | 56 (42.1%) | 27 (20.3%) | 16 (20.3%) |
| ... the opportunity to quit participation before the end of a trial without having disadvantages. | 45 (33.8%) | 45 (33.8%) | 24 (18.0%) | 19 (14.3%) |
| Sources of information on clinical trials used so far |  |  |  |  |
| So far, I have... | yes |  | no |  |
| ... used information provided by media / internet / journals. | 115 (87.1%) |  | 17 (12.9%) |  |
| ... received information from family members / friends. | 52 (39.4%) |  | 80 (60.6%) |  |
| received information from my local doctor(s) (e. g. general practitioner). | 63 (47.7%) |  | 69 (52.3%) |  |
| ... received information from my doctor(s) in hospital. | 101 (76.5%) |  | 31 (23.5%) |  |
|  | Fully applies | Rather applies | Does rather not apply | Does not apply at all |
| ... evaluated these references as trustful. | 57 (43.2%) | 64 (48.5%) | 8 (6.1%) | 3 (2.3%) |
| Study experience |  |  |  |  |
|  | yes |  | no |  |
| I have been offered the participation in a trial or had the opportunity to participate, respectively. | 95 (72.0%) |  | 37 (28.0%) |  |
| More than once I have been offered the participation in a trial or had the opportunity to participate, respectively. | 67 (70.5%) |  | 28 (29.5%) |  |
| My child/children has/have been offered the participation in a trial or had the opportunity to participate, respectively. | 32 (36.4%) |  | 56 (63.6%) |  |
| More than once my child/children has/have been offered the participation in a trial or had the opportunity to participate, respectively. | 19 (59.4%) |  | 13 (40.6%) |  |
| I know the cancer predisposition syndrome registry (CPS-registry Hannover/Heidelberg). | 114 (87.0%) |  | 17 (13.0%) |  |
| I know the survey on psychosocial aspects in LFS in Heidelberg or Hannover. | 80 (61.5%) |  | 50 (38.5%) |  |
| I know the whole-body-MRI study für affected with LFS in Heidelberg/Hannover. | 101 (77.7%) |  | 29 (22.3%) |  |
| I have already participated in a trial. | 82 (86.3%) |  | 13 (13.7%) |  |
| I have already participated in a trial more than once. | 59 (72.0%) |  | 23 (28.0%) |  |
| I am registered in the cancer predisposition syndrome registry (CPS-registry Hannover/Heidelberg). | 96 (85.0%) |  | 17 (15.0%) |  |
| I have already participated in the whole-body-MRI study für affected with LFS in Heidelberg/Hannover. | 69 (69.0%) |  | 31 (31.0%) |  |
| I have already participated in the survey on psychosocial aspects in LFS in Heidelberg or Hannover. | 60 (75.9%) |  | 19 (24.1%) |  |
| My child/children has/have already participated in a trial. | 23 (71.9%) |  | 9 (28.1%) |  |
| My child/children has/have already participated in a trial more than once. | 18 (78.3%) |  | 5 (21.7%) |  |
| My child/children is/are registered in the cancer predisposition syndrome registry (CPS-registry Hannover/Heidelberg). | 20 (90.9%) |  | 2 (9.1%) |  |
|  | Fully applies | Rather applies | Does rather not apply | Does not apply at all |
| In general, I would evaluate my experience with trials as „good“. | 43 (53.1%) | 37 (45.7%) | 1 (1.2%) | 0 (0.0%) |
| Attitudes toward clinical trials on LFS |  |  |  |  |
|  | Fully applies | Rather applies | Does rather not apply | Does not apply at all |
| I would approve cancer risk trials with adults with LFS. | 105 (82.0%) | 23 (18.0%) | 0 (0.0%) | 0 (0.0%) |
| I would approve cancer risk trials with children with LFS. | 87 (68.0%) | 37 (28.9%) | 3 (2.3%) | 1 (0.8%) |
| I believe that cancer risk studies with adults with LFS are too burdensome for them. | 5 (3.9%) | 14 (10.9%) | 74 (57.8%) | 35 (27.3%) |
| I believe that cancer risk studies with children with LFS are too burdensome for them. | 11 (8.6%) | 32 (25.0%) | 65 (50.8%) | 20 (15.6%) |
| I think I could contribute to medical improvements in LFS. | 90 (72.6%) | 34 (27.4%) | 0 (0.0%) | 0 (0.0%) |
| I think that my participation in a trial might help others who are affected with LFS. | 85 (68.5%) | 38 (30.6%) | 1 (0.8%) | 0 (0.0%) |
| I believe that I might receive a better treatment in a LFS trial. | 42 (33.9%) | 53 (42.7%) | 1 (0.8%) | 28 (22.6%) |
| I imagine to be supervised more closely during participation in a trial. | 33 (26.6%) | 67 (54.0%) | 19 (15.4%) | 5 (4.0%) |
| My family would support my participation in a trial. | 45 (36.3%) | 63 (50.8%) | 14 (11.3%) | 2 (1.6%) |
| I trust doctors of a trial’s team. | 63 (50.8%) | 57 (46.0%) | 4 (3.2%) | 0 (0.0%) |
| I am afraid of possible side-effects from medication that is being investigated in a trial. | 28 (22.6%) | 50 (40.3%) | 40 (32.3%) | 6 (4.8%) |
| I would feel like a guinea-pig. | 5 (4.0%) | 21 (16.9%) | 50 (40.3%) | 48 (38.7%) |
| I think that research only supports the investigators‘ carrier. | 1 (0.8%) | 4 (3.2%) | 36 (29.0%) | 83 (66.9%) |
| I would regret having participated in case the medication would not have the expected effect. | 7 (5.6%) | 13 (10.5%) | 77 (62.1%) | 27 (21.8%) |
| I am afraid of health-related disadvantages if I do not participate in an offered trial. | 13 (10.5%) | 24 (19.4%) | 54 (43.5%) | 33 (26.6%) |
| I would quit participation prematurely in case I found out that I would be randomized to the control group that does not receive the study medication. | 6 (4.8%) | 21 (16.9%) | 64 (51.6%) | 33 (26.6%) |
| Sources of information on clinical trials on LFS preferred in future |  |  |  |  |
| I would search for information about clinical trials via… | Fully applies | Rather applies | Does rather not apply | Does not apply at all |
| ... clinic websites. | 52 (39.4%) | 54 (40.9%) | 23 (17.4%) | 3 (2.3%) |
| ... the FIT-Website (www.krebs-praedisposition.de). | 65 (49.2%) | 47 (35.6%) | 14 (10.6) | 6 (4.5%) |
| ... the LFSA-Website (Li-Fraumeni Syndrome Association) or contact the LFSA Deutschland e. V. | 84 (63.6%) | 43 (32.6%) | 4 (3.0%) | 1 (0.8%) |
| ... support groups other than the LSFA. | 20 (15.2%) | 37 (28.0%) | 52 (39.4%) | 23 (17.4%) |
| ... social media (Facebook, Instagram, ...). | 25 (18.9%) | 45 (34.1%) | 38 (28.8%) | 24 (18.2%) |
| ... printed brochures or journals. | 20 (15.2%) | 49 (37.1%) | 40 (30.3%) | 23 (17.4%) |
| ... local doctors. | 32 (24.2%) | 52 (39.4%) | 35 (26.5%) | 13 (9.8%) |
| ... hospital doctors. | 62 (47.0%) | 61 (46.2%) | 5 (3.8%) | 4 (3.0%) |
| Participation in clinical trials (in general / on LFS) |  |  |  |  |
|  | Fully applies | Rather applies | Does rather not apply | Does not apply at all |
| In principle, I would participate in medical trials in general. | 63 (49.2%) | 45 (35.2%) | 16 (12.5%) | 4 (3.1%) |
| In principle, I would participate in a LFS trial. | 103 (80.5%) | 24 (18.8%) | 1 (0.8%) | 0 (0.0%) |
| In principle, I would let my child participate in medical trials in general. | 35 (27.3%) | 44 (34.4%) | 35 (27.3%) | 14 (10.9%) |
| In principle, I would let my child participate in a LFS trial. | 56 (43.8%) | 52 (40.6%) | 15 (11.7%) | 5 (3.9%) |
| Factors influencing willingness to participate |  |  |  |  |
|  | Fully applies | Rather applies | Does rather not apply | Does not apply at all |
| ... the trial’s aim would be to investigate a new medication’s effect in LFS. | 76 (59.4%) | 45 (35.2%) | 5 (3.9%) | 2 (1.6%) |
| ... the trial’s aim would be to investigate the tolerance of a new medication in LFS. | 63 (49.2%) | 50 (39.1%) | 13 (10.2%) | 2 (1.6%) |
| ... in the trial a new medication would be prescribed with the aim to decrease cancer risk in LFS. | 78 (60.9%) | 45 (35.2%) | 4 (3.1%) | 1 (0.8%) |
| … it was conducted internationally. | 53 (41.4%) | 49 (38.3%) | 21 (16.4%) | 5 (3.9%) |
| ... I would receive an expense allowance for it. | 11 (8.6%) | 17 (13.3%) | 61 (47.7%) | 39 (30.5%) |
| ... it would include coming to the clinics for study visits. | 7 (5.6%) | 20 (16.0%) | 62 (49.6%) | 36 (28.8%) |
| ... it would include additional blood collection. | 6 (4.8%) | 9 (7.2%) | 52 (41.6%) | 58 (46.4%) |
| ... I could have side effects from a medication I would take. | 24 (19.2%) | 52 (41.6%) | 38 (30.4%) | 11 (8.8%) |
| ... it would be possible that I receive a placebo. | 21 (16.8%) | 37 (29.6%) | 48 (38.4%) | 19 (15.2%) |
| ... I would be randomized to one out of two treatment options. | 11 (8.8%) | 41 (32.8%) | 54 (43.2%) | 19 (15.2%) |
| ... it would be possible that I would be part of a control group that does not receive the investigated medication. | 20 (16.0%) | 36 (28.8%) | 50 (40.0%) | 19 (15.2%) |
| Participation in Metformin Scenario |  |  |  |  |
|  | Fully applies | Rather applies | Does rather not apply | Does not apply at all |
| In principle, I would participate in the above-mentioned trial in the scenario. | 50 (41.3%) | 54 (44.6%) | 15 (12.4%) | 2 (1.7%) |
| I would wish children to participate in the trial in the scenario as well, so that – in the case of positive results - the medication could also be approved for children. | 38 (31.1%) | 58 (47.5%) | 21 (17.2%) | 5 (4.1%) |
| In principle, I would let my child participate in the above-mentioned trial in the scenario. | 38 (31.4%) | 47 (38.8%) | 27 (22.3%) | 9 (7.4%) |
| Factors influencing willingness to participate in Metformin Scenario |  |  |  |  |
| In principle, I would participate in the trial in the above-described scenario… | Fully applies | Rather applies | Does rather not apply | Does not apply at all |
| ... after having received more information about the study medication. | 53 (43.4%) | 58 (47.5%) | 10 (8.2%) | 1 (0.8%) |
| ... because I would hope for health advantages for myself. | 59 (48.4%) | 52 (42.6%) | 9 (7.4%) | 2 (1.6%) |
| ... because I might help other people with LFS by participating. | 79 (64.8%) | 39 (32.0%) | 4 (3.3%) | 0 (0.0%) |
| ... because I think that my quality of life might improve. | 39 (32.0%) | 54 (44.3%) | 22 (18.0%) | 7 (5.7%) |
| ... because I think that by participating I might have less fear of (further) occurence malignancies. | 50 (42.0%) | 43 (36.1%) | 17 (14.3%) | 9 (7.6%) |
| ... although the study medication might cause side-effects. | 27 (22.1%) | 55 (45.1%) | 36 (29.5%) | 4 (3.3%) |
| ... after being explained advantages and risks of participation. | 62 (50.8%) | 51 (41.8%) | 8 (6.6%) | 1 (0.8%) |
| I would not participate in the trial in the above-mentioned scenario… | Fully applies | Rather applies | Does rather not apply | Does not apply at all |
| ... because I am afraid of potential side-effects. | 18 (14.9%) | 29 (24.0%) | 50 (41.3%) | 24 (19.8%) |
| ... because I would not want to be in the control group at all. | 8 (6.6%) | 26 (21.5%) | 54 (44.6%) | 33 (27.3%) |
| ... if I would not receive an expense allowance. | 8 (6.6%) | (38.8%) | 66 (54.5%) | 0 (0.0%) |
| ... because I would not want to undergo blood collections. | 10 (8.3%) | 28 (23.1%) | 83 (68.6%) | 0 (0.0%) |
| ... because the way to the study center would be too time-consuming. | 8 (6.6%) | 29 (24.0%) | 49 (40.6%) | 35 (28.9%) |
| ... because I think that my quality of life would suffer from it. | 2 (1.7%) | 16 (13.2%) | 66 (54.5%) | 37 (30.6%) |
| ... because I think that my health would suffer from it. | 5 (4.1%) | (17.4%) | 67 (55.4%) | 28 (23.1%) |
